# Antiprotozoal Activity of Quinonemethide Triterpenes from *Maytenus ilicifolia* (Celastraceae)

**DOI:** 10.3390/molecules18011053

**Published:** 2013-01-15

**Authors:** Vania A. F. F. M. dos Santos, Karoline M. Leite, Mariana da Costa Siqueira, Luis O. Regasini, Isabel Martinez, Camila T. Nogueira, Mariana Kolos Galuppo, Beatriz S. Stolf, Ana Maria Soares Pereira, Regina M. B. Cicarelli, Maysa Furlan, Marcia A. S. Graminha

**Affiliations:** 1 Instituto de Quimica, Universidade Estadual Paulista, UNESP, PO BOX 355, Araraquara-SP 14801-970, Brazil; 2 Faculdade de Ciencias Farmaceuticas, Universidade Estadual Paulista, UNESP, Araraquara-SP 14801-902, Brazil; 3 Instituto de Ciencias Biomedicas, Universidade de São Paulo, São Paulo 05508-000, Brazil; 4 Departamento de Biotecnologia Vegetal, Universidade de Ribeirão Preto, Ribeirão Preto-SP 14096-900, Brazil

**Keywords:** Celastraceae, *Maytenus ilicifolia*, leishmanicidal, trypanocidal, quinone-methide triterpenes, maytenin, pristimerin

## Abstract

The present study describes the leishmanicidal and trypanocidal activities of two quinonemethide triterpenes, maytenin (**1**) and pristimerin (**2**), isolated from *Maytenus ilicifolia* root barks (Celastraceae). The compounds were effective against the Trypanosomatidae *Leishmania amazonensis* and *Leishmania chagasi* and *Trypanosoma cruzi*, etiologic agents of leishmaniasis and Chagas’ disease, respectively. The quinonemethide triterpenes **1** and **2** exhibited a marked *in vitro* leishmanicidal activity against promastigotes and amastigotes with 50% inhibitory concentration (IC_50_) values of less than 0.88 nM. Both compounds showed IC_50_ lower than 0.3 nM against *Trypanosoma cruzi* epimastigotes. The selectivity indexes (SI) based on BALB/c macrophages for *L. amazonensis* and *L. chagasi* were 243.65 and 46.61 for (**1**) and 193.63 and 23.85 for (**2**) indicating that both compounds presented high selectivity for *Leishmania* sp. The data here presented suggests that these compounds should be considered in the development of new and more potent drugs for the treatment of leishmaniasis and Chagas’ disease.

## 1. Introduction

The World Health Organization (WHO) estimates that more than one billion people suffer from neglected tropical diseases. Leishmaniasis is a widespread disease, affecting 12 million people around the world with about 1–2 million estimated new cases occurring every year [1]. About 8–10 million people are infected with *Trypanosoma cruzi* (the parasite that causes Chagas’ disease), mostly in Latin America [2].

For more than 60 years, pentavalent antimonials, Sb (V) compounds, have been the first-line chemotherapy against leishmaniasis, but the emergence of resistant strains has limited their usefulness. Alternatively, amphotericin B, pentamidine isethionate, miltefosine and paramomycin are available, but their use is limited due to toxicity or high cost of treatment [3]. Benznidazole is the only drug manufactured for the treatment of Chagas’ disease, but is only effective during the acute phase of the disease [4,5]. Searching for new drugs with high and specific activity is very important, especially in countries where these parasitic diseases constitute a serious public health problem [6].

The existing Brazilian biodiversity is a potential source of many unknown bioactive molecules [7] that may be studied in search for natural products with leishmanicidal and trypanocidal activities.

The friedo-nor-oleanane derivatives are members of a small group of triterpenoid natural products known as quinonemethides and are considered as chemotaxonomic indicators of the Celastraceae family [8]. They accumulate in the stem bark and root barks of some family species [9]. Many biological activities have been reported for members of this class, including antitumor [10], antioxidant [11,12] and trypanocidal [13]. Here, we present the effect of two quinonemethide triterpenes, maytenin (**1**) and pristimerin (**2**) [14], on the viability of *Leishmania* promastigotes and intracellular amastigotes and of *T. cruzi* epimastigotes. These compounds were isolated from the roots of the Brazilian plant Espinheira santa.

## 2. Results and Discussion

### 2.1. Maytenin *(**1**)* and Pristimerin *(**2**):* Leishmanicidal, Trypanocidal and Cytotoxic Activities against Extracellular Parasites and Macrophages

New potential leishmanicidal and trypanocidal drugs are generally screened *in vitro* against promastigote and epimastigote forms [15]. Although the most clinically relevant stages of Trypanosomatidae parasites are intracellular amastigotes and trypomastigotes, extracellular promastigotes and epimastigotes have the obvious advantage of being easier and cheaper to handle in the large-scale requirement of high throughput screening methods. Furthermore, promastigotes and epimastigotes have common metabolic machinery and pathways, and potential therapeutic targets effective against these extracellular forms may be relevant against the intracellular ones [16]. One of the most commonly used methods in drug screening for parasitic diseases is the microscopic counting of viable parasites, which is time-consuming, labor-intensive, and dependent on the ability of the observer [17]. For this reason, we decided to carry out a colorimetric viability assay using the tetrazolium salt MTT, which is a rapid, simple, and reliable method for evaluation of leishmanicidal and trypanocidal activities [15].

**Figure 1:**
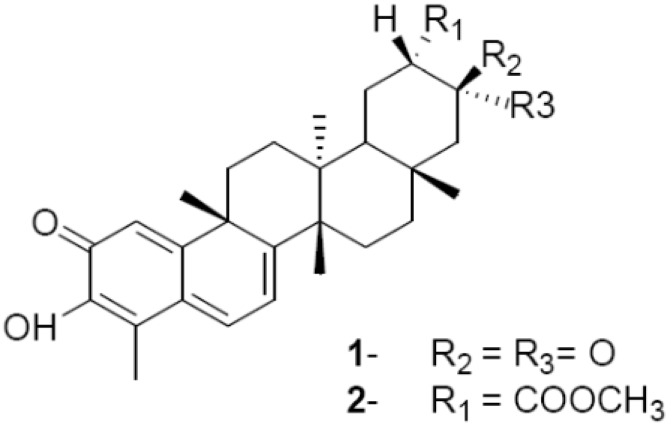
Chemical structures of compounds **1** and **2** from *M. ilicifolia*.

The quinonemethide triterpenes are among the secondary metabolites of interest since many of them exhibit a broad range of biological activities. In the present study we observed that compounds **1** and **2** (Figure 1) showed potent *in vitro* activity against *Leishmania amazonensis* and *Leishmania chagasi* promastigotes and *Trypanosoma cruzi* epimastigotes. As reported in Table 1, the IC_50_ values obtained for compounds (**1**) and (**2**) were 0.09 nM and 0.05 nM for *L. amazonensis* promastigotes and 0.46 nM and 0.41 nM for *L. chagasi* promastigotes, respectively. The IC_50_ values for *T. cruzi* epimastigotes were 0.25 nM and 0.30 nM, respectively. The two compounds showed potent activity when compared to the positive controls pentamidine for *L. amazonensis* (IC_50_ = 6.75 nM) and *L. chagasi* (IC_50_ = 4.0 nM) and benznidazole for *T. cruzi* (IC_50_ = 31.20 μM). The selectivity index (SI), a relevant characteristic for defining hit compounds [18], was calculated for compounds **1** and **2** by dividing their cytotoxic activity against murine macrophages (LC_50_) by their leishmanicidal and trypanocidal activities (SI = LC_50_/IC_50_). Macrophages were employed for SI calculation since they are the main host cell for *Leishmania* and because they were used for the intracellular experiments. Using the LC_50_ values of 21.25 nM and 9.71 nM, which were found for murine macrophages treated with compounds **1** and **2**, respectively, we obtained SIs for *L. amazonensis* and *L. chagasi* of 243.65 and 46.61 for **1** and 193.63 and 23.85 for **2**, as shown in Table 1. For *T. cruzi* epimastigotes the SIs were 85 for **1** and 332.37 for **2**. These results indicate that both compounds have good selectivity for trypanosomatid extracellular forms. It is interesting to note that *L. amazonensis* presented much higher sensitivity (five and eight times higher) to both compounds when compared to *L. chagasi*. Variations in sensitivity to several drugs among promastigotes of different species have also been reported in previous studies. Given the known biochemical and molecular differences between *Leishmania* species, it is not surprising that there is variation in intrinsic sensitivity to several drugs [19]. In fact, sensitivities to miltefosine varied among promastigotes of *Leishmania donovani*, *Leishmania major*, *Leishmania tropica*, *Leishmania aethiopica*, *Leishmania mexicana*, and *Leishmania panamensis*. *L.donovani* was the most sensitive species, whereas *L. major* was the least sensitive species [20]. On the other hand, promastigotes of *L. major* were the most sensitive to tamoxifen [21].

**Table 1:** Leishmanicidal, trypanocidal and cytotoxic activities of maytenin (**1**) and pristimerin (**2**) in nanomolar.

compound	*L. amazonensis* (IC_50_)		*L. chagasi* (IC_50_)		*T. cruzi* (IC_50_)	BALB/c (LC_50_)
	promastigotes	amastigotes	promastigotes	amastigotes	epimastigotes	macrophages
maytenin	0.09 (243.65)	0.47 (45.21)	0.46 (46.41)	0.25 (85)	0.25 (85)	21.25
pristimerin	0.05 (193.63)	0.88 (11.03)	0.41 (23.85)	0.43 (22.65)	0.30 (32.37)	9.71
control	6.75 ^a^ (3.0)	9.30 (2.18)	6.75 ^a^	n.d.	31.20 × 10^3^	20.32 ^a^

All IC_50_/LC_50_ values are in nM and selectivity indices (SI) are shown in parentheses; a, pentamidine; b, benznidazol; n.d. not determined.

### 2.2. Leishmanicidal Activity of Compounds ***1*** and ***2*** on Intracellular Parasites

**Figure 2:**
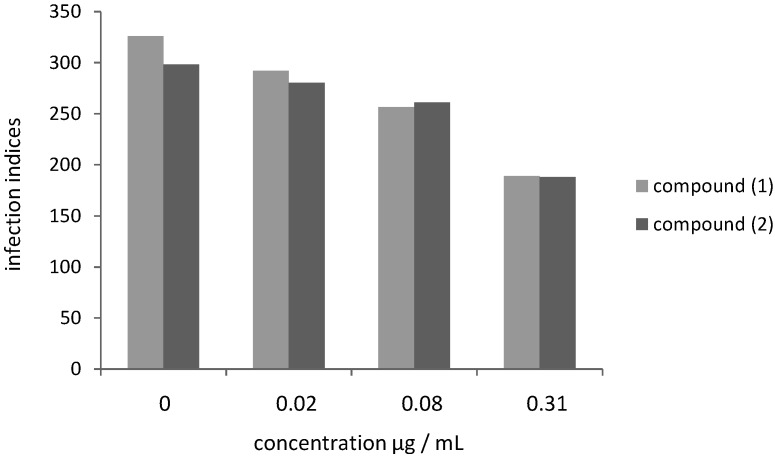
Effect of compounds **1** and **2** on *L. amazonensis* infection of peritoneal BALB/c macrophages.

**Figure 3:**
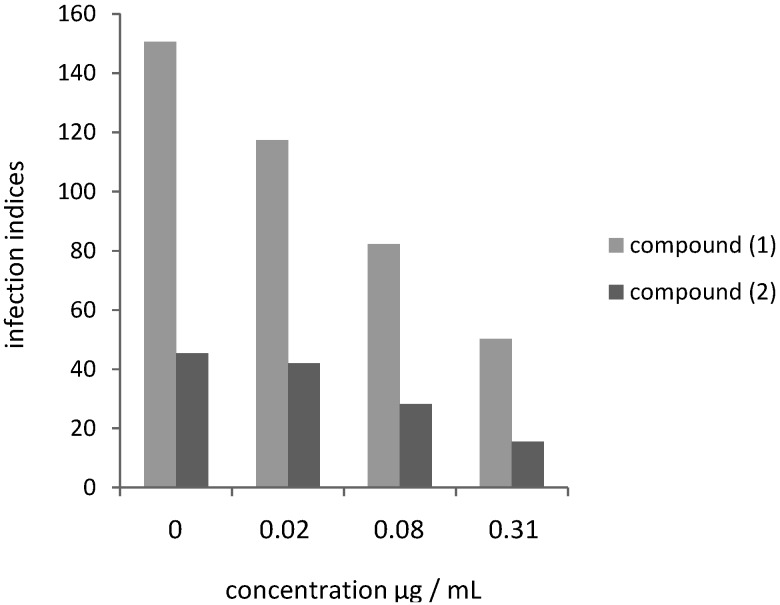
Effect of compounds **1** and **2** on *L. chagasi* infection of peritoneal BALB/c macrophages.

Subsequently, we analyzed whether the quinonemethide triterpenes **1** and **2** had antiprotozoal activity against *L. amazonensis* and *L. chagasi* intracellular amastigotes. When infected macrophages were treated with compounds (**1**) and (**2**), a dose-dependent decrease in the infection indices was observed. The effects of the drugs on *Leishmania* infection indices were calculated based on three different drug concentrations (0.02, 0.08 and 0.31 µg/mL), as shown in Figure 2 and Figure 3.

For *L. amazonensis*, the results presented in Figure 2 correspond to compounds **1** and **2** cytotoxicity indices of 10.43, 21.47, 42.02% and 6.03, 12.50, 36.98%, respectively. For *L. chagasi*, the results shown in Figure 3 correspond to compounds **1** and **2** cytotoxicity indices of 22.01, 45.32, 66.58% and 7.28, 37.53, 65.78%, respectively. The corresponding IC_50_ values for **1** and **2** were calculated for amastigotes of *L. amazonensis* (0.47 nM/0.88 nM) and *L. chagasi* (0.25 nM/0.43 nM) (Table 1). Contrarily to the observed for promastigote forms, amastigotes of *L. amazonensis* were more resistant to both compounds when compared to *L. chagasi* (Table 1), although the differences were less pronounced for the intracellular forms than for the promastigotes. Variations in susceptibility to drugs among *Leishmania* species have been previously reported using amastigote-macrophage model. *L. donovani* and *Leishmania braziliensis* amastigotes were found to be three to fivefold more sensitive to sodium stibogluconate than *L. major*, *L. tropica*, and *L. Mexicana* [19]. Using the same model, *L. mexicana* amastigotes were the least sensitive to amphotericin B among six species, possibly due to variation**s** in the type and quantity of sterols, the targets of this drug [20].

## 3. Experimental

### 3.1. General Methods

TLC analyses were performed using Merck silica gel 60 (230 mesh) and precoated silica gel 60 PF_254_ Analytical HPLC-DAD was performed on a Varian Pro Star 240, with a diode array ultraviolet detector, and the separation was achieved using a reverse phase in a Phenomenex C_18_ column (250 mm × 4.6 mm). Preparative HPLC was performed on a Varian Prep-Star 400 system using a reverse phase Phenomenex C_18_ (250 mm × 21.2 mm) preparative column. 1D (^1^H, ^13^C, DEPT 90° and DEPT 135°) and 2D gHMQC, gHMBC and ^1^H-^1^H gCOSY) NMR experiments were recorded on a Varian INOVA 500 spectrometer (11.7 T) at 500 MHz (^1^H) and 125 MHz (^13^C), respectively, with CDCl_3_ as internal reference. Positive-ion low-resolution mass spectra were acquired on an MS ultrOTOFQ-ESI-TOF instrument (Bruker Daltonics, Raleigh, NC, USA), using MeOH-H_2_O (1:1) as solvent and a cone voltage of 40 V.

### 3.2. Plant Material

Three-years-old root barks of *Maytenus ilicifolia* was collected in district of Juruce, municipality of Jardinopolis (São Paulo State, Brazil, at 21°04'18.8'' S; 47°44'08.8'' W) in March 2010. The plant was identified by Rita Maria de Carvalho. A voucher specimen (HPM-BR 0059) has been deposited in the Herbarium of the University of Campinas, São Paulo, Brazil.

### 3.3. Extraction and Isolation of Quinonemethide Triterpenes from Maytenus ilicifolia

Roots (270.00 g) were frozen in liquid N_2_ and mechanically pulverized in a mortar. Afterwards, the material was submitted to extraction in ultrasonic bath using dichloromethane. Solvent was removed by distillation under reduced pressure, providing a dichloromethane extract (4.9 g). An aliquot of 1.0 g of crude extract was fractionated by five 5 fractions were obtained (F1–5). The extract was then fractionated by prep. TLC and eluted with hexane-EtOAc (8:2), whereupon five fractions were obtained. After being analyzed by HPLC-DAD, fractions F2 (280 mg) and F3 (240 mg) were submitted to preparative HPLC eluted with MeOH-H_2_O (9:1, flow rate: 10 mL min^−1^; detection: 254 nm) and MeOH-H_2_O (8:2, flow rate: 12 mL min^−1^; detection: 254 nm), respectively, generating **1** (59.0 mg) and **2** (20.6 mg). Compounds **1** and **2** were characterized by comparing their ^1^H-NMR, ^13^C-NMR, ES-MS, and the assignments were based on 2D-NMR experiments, including gHMQC and gHMBC, and were compared with literature values [22].

*Maytenin* (**1**). Reddish-brown amorphous solid; EIMS *m*/*z* [M+1]^+^ 421.30 (calculated for C_28_H_36_O_3_), ^1^H-NMR (CDCl_3_, 500 MHz): δ = 0.92 (3H, s, CH_3_-27), 0.95 (3H, s, CH_3_-30), 1.28 (3H, s, CH_3_-26), 0.94 (3H, s, CH_3_-28), 2.84 (1H, brd, *J =* 14.5 Hz, H-22β), 1.45 (3H, s, CH_3_-25),1.79 (1H, d, *J* = 14.5 Hz, H-22α), 2.16 (3H, s, CH_3_-23), 6.31 (1H, d, *J* = 7.5 Hz, H-7), 6.48 (1H, d, *J* = 1.5 Hz, H-1), 6.97 (1H, dd, *J* = 7.2 and 1.0 Hz, H-6). ^13^C-NMR (CDCl_3_, 500 MHz): δ = 10.24 (C-23), 15.06 (C-30), 19.70 (C-27), 21.54 (C-26), 32.05 (C-19), 28.50 (C-15), 29.93 (C-12), 38.17 (C-17), 40.62 (C-20), 33.78 (C-11), 32.54 (C-28), 35.50 (C-16), 39.03 (C-25), 40.62 (C-13), 42.70 (C-9), 43.53 (C-18), 52.52 (C-22), 44.64 (C-14), 213.54 (C-21), 117.15 (C-4), 118.12 (C-7), 119.77 (C-1), 127.74 (C-5), 133.57 (C-6), 146.08 (C-3), 164.69 (C-10), 168.64 (C-8), 178.40 (C-2).

*Pristimerin* (**2**). Orange-red amorphous solid; EIMS *m*/*z* [M+1]^+^ 465.30 (calculated for C_30_H_40_O_4_), ^1^H-NMR (CDCl_3_, 500 MHz): δ = 0.47 (3H, s, CH_3_-27), 1.11 (3H, s, CH_3_-30), 1.19 (3H, s, CH_3_-26), 1.03 (3H, s, CH_3_-28), 1.38 (3H, s, CH_3_-25), 2.14 (3H, s, CH_3_-23), 6.28 (1H, d, *J* = 7.0 Hz, H-7), 6.47 (1H, d, *J* = 1.5 Hz, H-1), 6.94 (1H, dd, *J* = 7.0 and 1.5 Hz, H-6), 3.48 (3H, s, CH_3_O-29). ^13^C-NMR (CDCl_3_, 500 MHz): δ = 10.22 (C-23), 32.36 (C-30), 18.32 (C-27), 21.62 (C-26), 30.90 (C-19), 28.65 (C-15), 29.66 (C-12), 30.53 (C-17), 40.41 (C-20), 33.57 (C-11), 31.58 (C-28), 36.39 (C-16), 38.25 (C-25), 39.42 (C-13), 42.93 (C-9), 44.32 (C-18), 34.80 (C-22), 45.04 (C-14), 29.88 (C-21), 117.10 (C-4), 118.12 (C-7), 119.56 (C-1), 127.43 (C-5), 134.02 (C-6), 146.02 (C-3), 164.76 (C-10), 170.03 (C-8), 178.34 (C-2), 51.53 (CH_3_O).

### 3.4. Parasite Culture

Promastigotes of *L. amazonensis* MPRO/BR/1972/M1841-LV-79, *L. chagasi* MHOM/BR/1974/PP75 strains and epimastigotes of *T. cruzi* Y strain were maintained at 28 °C in liver-infusion Tryptose (LIT) supplemented with 10% fetal calf serum (FCS).

### 3.5. Evaluation of Leishmanicidal Activity

#### 3.5.1. Promastigotes

Cultured promastigotes at the end of the exponential growth phase were seeded at 8 × 10^6^ parasites/mL in 96 well flat-bottom plates (Costar^®^). Maytenin and pristimerin were dissolved in DMSO (the highest concentration was 1.4%, which was not hazardous to the parasites, as previously accessed), added to parasite suspension to final concentrations between 1.6 µg/mL and 100 µg/mL and incubated at 28 °C for 72 h. The assays were carried out in triplicate. Pentamidine isethionate purchased from Sigma-Aldrich (St Louis, MO, USA) was used as reference drug (a 16.7 mg/mL stock solution was prepared in sterile deionized water) and added to parasite suspension to final concentrations between 1.6 and 100 µg/mL. Leishmanicidal effects were assessed by 3-[4,5-dimethylthiazol-2-yl]-2,5-diphenyltetrasodium bromide (MTT) method with modifications [23]. Absorbances were read at 490 nm. The drug concentration corresponding to 50% of parasite growth inhibition was expressed as the inhibitory concentration (IC_50_).

#### 3.5.2. Cytotoxicity on Macrophages

The cytotoxicity was evaluated on mouse peritoneal macrophages. Peritoneal macrophages were collected from BALB/c mice and were seeded in 96 well flat-bottom plates (Costar^®^) at a density of 1 × 10^5^ cells/well (100 µL/well) in RPMI 1640 medium supplemented with 10% heat inactivated fetal calf serum, 25 mM HEPES and 2 mM L-glutamine and incubated for 24 h at 37 °C in a 5% CO_2_-air mixture. The medium was then removed and the cells were treated with different concentrations of quinonemethides triterpenes. Positive (with pentamidine) and negative (without drugs) controls were included in each experiment. The plates were incubated under the same conditions for 24 h. Subsequently, the MTT colorimetric assay was carried out as described above. Absorbance was read in a 96-well plate reader (Robonik^®^) at 595 nm. The 50% lethal concentration (LC_50_) was determined by logarithm regression analysis of the obtained data. The cytotoxicity for macrophages and for promastigotes was compared using the selectivity index (SI), which was determined as the ratio between LC_50_ for macrophages and IC_50_ for promastigotes.

#### 3.5.3. Amastigotes

Peritoneal macrophages from BALB/c mice were plated at 3 × 10^5^ cells/well on coverslips (13 mm diameter) previously arranged in a 24-well plate in RPMI 1640 medium supplemented with 10% inactivated FCS, and allowed to adhere for 4 h at 37 °C in 5% CO_2_. Adherent macrophages were infected with promastigotes in the stationary growth phase using a ratio of 5:1 (parasites:cell) at 37 °C in 5% CO_2_ for 4 h. After that time, the non-internalized parasites were removed by washing, and infected cultures were incubated in RPMI medium or treated with different concentrations of quinonemethide triterpenes for 24 h. The cells were then fixed in a methanol solution and stained with Giemsa. The infection index was determined by multiplying the percentage of infected macrophages by the mean number of amastigotes per infected cell. The concentration that caused a 50% decrease in the infection index compared to the control was determined by regression analysis of the log-transformed data. All experiments involving mice were performed as indicated by the ethical committee of Faculdade de Ciências Farmacêuticas, Universidade Estadual Paulista, Araraquara (protocol number CEUA/FCF/Car n°24/2012).

### 3.6. Evaluation of Trypanocidal Activity

All experiments were performed with *T. cruzi* Y strain epimastigote forms. The parasites were grown axenically at 28 °C in LIT medium supplemented with 10% fetal calf serum and harvested during the exponential growth phase (7-day-old culture forms). It was added 1 × 10^7^ parasites/mL to each well of a 96-well microplate and the same volume of LIT medium was used as control. Compounds were dissolved in dimethyl sulfoxide (DMSO) and further added to the microplate for final concentrations from 1 μg/mL to 100 μg/mL. The plates were maintained at 28 °C for 72 h and MTT was performed as described above. MTT assay was prepared as described elsewhere [15]. Absorbance of samples was read at 595 nm. All assays were conducted in triplicate. The drug concentration corresponding to 50% of parasite growth inhibition was expressed as the IC_50_ and was determined from sigmoidal regression of the concentration-response curves after 72 h of incubation (Benznidazole was employed as positive control). For the statistical analysis Probit´s method was employed [24].

## 4. Conclusions

The results herein presented suggest that the quinonemethide triterpenes **1** and **2** should be further considered in the development of new and more potent drugs for the treatment of leishmaniasis and Chagas’ disease.

## References

[1] Alvar J., Vélez I.D., Bern C., Herrero M., Desjeux P., Cano J., Jannin J., den Boer M., Team W.L.C. (2012). Leishmaniasis worldwide and global estimates of its incidence. PLoS One.

[2] Rassi A., de Rezende M. (2012). Leishmaniasis worldwide and global estimates of its incidence. Infect. Dis. Clin. N. Am..

[3] Chawla B., Madhubala R. (2010). Drug targets in *Leishmania*. J. Parasit. Dis..

[4] Vinhaes M.C., Dias J.C. (2000). Chagas disease in Brazil. Cad. Saude Publica.

[5] Croft S.L., Brun R., Fairlamb A.H., Ridley R.G., Vial H.J. (2003). *In Vitro* and *in Vivo* Models for the Identification and Evaluation of Drugs Active against *Trypanosoma* and *Leishmania*. Drugs against Parasitic Diseases: R&D Methodologies and Issues (Discoveries and Drug Development).

[6] Da Silva Mota J., Leite A.C., Batista J.M., Noelí López S., Luz Ambrósio D., Duó Passerini G., Kato M.J., da Silva Bolzani V., Barretto Cicarelli R.M., Furlan M. (2009). *In vitro* trypanocidal activity of phenolic derivatives from *Peperomia obtusifolia*. Planta Med..

[7] De Mesquita M., Desrivot J., Bories C., Fournet A., de Paula J., Grellier P., Espindola L. (2005). Antileishmanial and trypanocidal activity of Brazilian Cerrado plants. Mem. Inst. Oswaldo Cruz.

[8] Truiti M.C., Ferreira I.C., Zamuner M.L., Nakamura C.V., Sarragiotto M.H., Souza M.C. (2005). Antiprotozoal and molluscicidal activities of five Brazilian plants. Braz. J. Med. Biol. Res..

[9] Gunatilaka A.A.L. (1996). Triterpenoid Quinonemethides and Related Compounds (Celastroloids).

[10] Corsino J., de Carvalho P.R., Kato M.J., Latorre L.R., Oliveira O.M., Araújo A.R., Bolzani V.D., França S.C., Pereira A.M., Furlan M. (2000). Biosynthesis of friedelane and quinonemethide triterpenoids is compartmentalized in *Maytenus aquifolium* and *Salacia campestri*. Phytochemistry.

[11] Peng B., Xu L., Cao F., Wei T., Yang C., Uzan G., Zhang D. (2010). HSP90 inhibitor, celastrol, arrests human monocytic leukemia cell U937 at G0/G1 in thiol-containing agents reversible way. Mol. Cancer.

[12] Dos Santos V.A., Dos Santos D.P., Castro-Gamboa I., Zanoni M.V., Furlan M. (2010). Evaluation of antioxidant capacity and synergistic associations of quinonemethide triterpenes and phenolic substances from *Maytenus ilicifolia* (Celastraceae). Molecules.

[13] Lião L.M., Silva G.A., Monteiro M.R., Albuquerque S. (2008). Trypanocidal activity of quinonemethide triterpenoids from *Cheiloclinium cognatum* (Hippocrateaceae). Z. Naturforsch. C.

[14] Goijman S.G., Turrens J.F., Marini-Bettolo G.B., Stoppani A.O. (1984). Inhibition of growth and macromolecular biosynthesis in *Trypanosoma cruzi* by natural products. Effects of miconidine and tingenone. Medicina (B Aires).

[15] Muelas-Serrano S., Nogal-Ruiz J.J., Gómez-Barrio A. (2000). Setting of a colorimetric method to determine the viability of *Trypanosoma cruzi* epimastigotes. Parasitol. Res..

[16] Siqueira-Neto J.L., Song O.R., Oh H., Sohn J.H., Yang G., Nam J., Jang J., Cechetto J., Lee C.B., Moon S. (2010). Antileishmanial high-throughput drug screening reveals drug candidates with new scaffolds. PLoS Negl. Trop. Dis..

[17] Le Fichoux Y., Rousseau D., Ferrua B., Ruette S., Lelièvre A., Grousson D., Kubar J. (1998). Short- and long-term efficacy of hexadecylphosphocholine against established *Leishmania infantum* infection in BALB/c mice. Antimicrob. Agents Chemother..

[18] Bollini M., Casal J.J., Alvarez D.E., Boiani L., González M., Cerecetto H., Bruno A.M. (2009). New potent imidazoisoquinolinone derivatives as anti-*Trypanosoma cruzi* agents: Biological evaluation and structure-activity relationships. Bioorg. Med. Chem..

[19] Croft S.L., Sundar S., Fairlamb A.H. (2006). Drug resistance in leishmaniasis. Clin. Microbiol. Rev..

[20] Escobar P., Matu S., Marques C., Croft S.L. (2002). Sensitivities of *Leishmania* species to hexadecylphosphocholine (miltefosine), ET-18-OCH(3) (edelfosine) and amphotericin B. Acta Trop..

[21] Miguel D.C., Yokoyama-Yasunaka J.K., Andreoli W.K., Mortara R.A., Uliana S.R. (2007). Tamoxifen is effective against *Leishmania* and induces a rapid alkalinization of parasitophorous vacuoles harbouring *Leishmania* (*Leishmania*) *amazonensis* amastigotes. J. Antimicrob. Chemother..

[22] Gunatilaka A.A.L., Fernando C.H., Kikuchi T., Tezuka Y. (1989). ^1^H and ^13^C-NMR analysis of three quinone-methide triterpenoids. Magn. Reson. Chem..

[23] Santos V.A., Regasini L.O., Nogueira C.R., Passerini G.D., Martinez I., Bolzani V.S., Graminha M.A., Cicarelli R.M., Furlan M. (2012). Antiprotozoal sesquiterpene pyridine alkaloids from Maytenus ilicifolia. J. Nat. Prod..

[24] Jeller A.H., Silva D.H., Lião L.M., Bolzani V.A.S., Furlan M. (2004). Antioxidant phenolic and quinonemethide triterpenes from *Cheiloclinium cognatum*. Phytochemistry.

